# The Prevalence, Magnitude, and Reversibility of Elevated Liver Enzyme Activities in Hyperthyroid Cats Presenting for Iodine-131 Treatment

**DOI:** 10.3389/fvets.2022.830287

**Published:** 2022-02-16

**Authors:** Joseph Campbell, Peter Chapman, Alan Klag

**Affiliations:** BluePearl Veterinary Specialty and Emergency Center, Levittown, PA, United States

**Keywords:** feline hyperthyroidism, liver enzyme elevations, I-131 treatment, clinical pathology data, thyroid nodular hyperplasia

## Abstract

**Objectives:**

The primary objective of this study was to report the prevalence and magnitude of elevated liver enzyme activity in feline hyperthyroidism using a large cohort of cats presenting for iodine-131 treatment. The secondary objective was to determine if elevated liver enzyme activity was a reversible process following successful iodine-131 treatment.

**Methods:**

Cases that presented for a single iodine-131 treatment were retrospectively reviewed. Short-term and long-term follow-up clinicopathologic data was then reviewed for the secondary objective.

**Results:**

Two hundred seventeen hyperthyroid cats met the inclusion criteria for the primary objective. In total, 123/217 (56.7%) of the cats had at least one liver enzyme elevation on their chemistry panel, with alanine transaminase activity being the most common. All cats who were successfully treated with iodine-131 had liver enzyme activity within the reference range at short-term follow-up and long-term follow-up points.

**Conclusion and Relevance:**

Our study demonstrates that elevated liver values are common in cats presenting for iodine-131 treatment. Additionally, our study demonstrates that even when liver values are markedly elevated prior to treatment, the liver enzyme activity will return to normal after successful resolution of hyperthyroidism using iodine-131 treatment. Investigation into hepatobiliary disease and liver function tests for cats with a diagnosis of hyperthyroidism may be unnecessary as the liver values will likely return to normal with successful iodine-131 treatment.

## Introduction

Feline hyperthyroidism is a widely recognized disease that is characterized by functional thyroid nodular hyperplasia or neoplasia (1–3). Although several treatment options exist, radioactive iodine-131 (I-131) therapy has a high success rate for resolution of clinical signs and decrease of thyroid hormone levels and has minimal side effects (4–8).

Among the clinicopathological findings in feline hyperthyroidism, elevated liver enzymes (ELEZ) have been reported up to 75% of pretreatment cats with hyperthyroidism; however, the cause of ELEZ in feline hyperthyroidism is unknown (9). Mitochondrial-induced apoptosis, oxidative deoxyribonucleic acid damage, or bone turnover in the thyrotoxic state has been proposed as possible causes for ELEZ in hyperthyroid cats (10–13). In human thyrotoxicosis, ELEZ are common, with up to 76% of patients having at least one liver function test abnormality, and the ELEZ are often reversible once the patient achieves a euthyroid state, and there is also no correlation with histopathologic lesions and ELEZ (14). One study with 19 hyperthyroid cats treated with I-131 could not demonstrate an association with abnormalities in the sonographic appearance of the hepatic parenchyma or liver functional variables (serum bile acids, albumin, ammonia, cholesterol, and blood urea nitrogen) in hyperthyroid cats regardless of the degree of ELEZ (78.94% of the cats had ELEZ), and the liver enzymes (LEZ) in these cats after I-131 therapy had no significant difference with age-matched healthy control cats (15).

The significance of ELEZ in feline hyperthyroidism is unknown and may represent a concomitant finding that may resolve with successful treatment of feline hyperthyroidism, or a separate entity that may require additional investigation and treatment. Liver histopathology of cats with hyperthyroidism usually show mild non-specific changes but can show some centrilobular fatty infiltration, like human thyrotoxicosis, in severely affected cats (1, 2, 9). Liver related concerns just before death have been previously reported in up to 2% of hyperthyroid cats; however, these concerns were stated as being mild clinical abnormalities and were not clearly defined (16).

A few studies demonstrated ELEZ in feline hyperthyroidism prior to other treatments followed by improvement in liver enzymes (LEZ) after successful treatment (ex: iodine-restricted diets, oral carbimazole, oral methimazole, methimazole transdermal); however, these studies were small with a limited number of cases (8–45 cats), many of the cats continued to have ELEZ after treatment, the study objectives did not include ELEZ, and the treatments in these studies did not include I-131 therapy (17–20).

The primary objective of this study was to report the prevalence and magnitude of ELEZ in feline hyperthyroidism using a large cohort of cats presenting for I-131 treatment. The secondary objective was to determine if ELEZ was a reversible process following successful I-131 treatment.

## Materials and Methods

### Case Selection

Records dated between November 2012 and December 2017 at Veterinary Specialty and Emergency Center in Levittown, Pennsylvania were retrospectively searched for cats receiving I-131 for treatment of feline hyperthyroidism.

Cases were included if they met the criteria for feline hyperthyroidism diagnosis and if they underwent one single I-131 treatment. Diagnosis of feline hyperthyroidism was established by the referring veterinarian as compatible clinical signs such as weight loss despite a good appetite, polyphagia, hyperactivity, and/or a palpable thyroid nodule or goiter in addition to elevated thyroxine concentration above the upper limit of the reference range for the analyzers used (IDEXX Laboratory 0.8–4.7 mcg/dl; Antech Laboratory 0.8–4.0 mcg/dl) based on previously established clinical criteria and clinicopathologic abnormalities (9). All cats received a physical exam including palpation for a thyroid nodule and assessment of approximate thyroid nodule size by a single clinician (Alan Klag). The dose of I-131 used was determined by a single clinician (Alan Klag) using a sliding scale based on the original thyroxine concentration at the time of diagnosis minus 5–10% of the calculated dose (T4: <7 mcg/dl, 3.5 mCi; 7–15 mcg/dl, 4.0 mCi; 15–20 mcg/dl, 4.5 mCi; and >20 mcg/dl, 5.0 mCi). The dose was sometimes reduced further at the discretion of the clinician if there were evidence of early kidney disease or mild azotemia (blood urea nitrogen or BUN, creatinine). Successful treatment was defined as improvement or resolution in clinical signs and a thyroxine concentration within reference range on the analyzer used. Treatment failure was defined as cats with clinical signs consistent with hyperthyroidism and thyroxine concentrations above the upper limit of the reference range on the analyzer used.

Exclusion criteria were incomplete records, inability to calculate magnitude of ELEZ due to lack of reference ranges, initial treatment failure requiring more than one I-131 treatment, cats receiving thyroperoxidase inhibitors or iodine restricted diets at the time of biochemistry data collection, and cats with evidence of possible liver failure or significant hepatobiliary disease, defined by a total bilirubin >1.0 mg/dl or an elevated gamma-glutanyl transferase (GGT) activity above the reference range.

### Data Collected

Data collected were complete blood count (CBC), serum biochemistry, thyroxine concentration, urinalysis, and radiographic imaging of the thorax and abdomen collected within 6 weeks from receiving I-131 treatment. CBC results were within normal limits and had no significant findings. Severe abnormalities on the CBC or severe azotemia on serum biochemistry profiles precluded consideration for I-131 treatment. Serum biochemistry profiles from various analyzers were used depending on the practice submitting the data for referral. Most of the serum biochemistry profiles were run on one of two analyzers at two large commercial laboratory institutions (Antech, *n* = 126; IDEXX, *n* = 73) with established reference ranges. The remaining serum biochemistry profiles were run on in-house analyzers (IDEXX, *n* = 13; Abaxis, *n* = 3; or Heska, *n* = 2), and those machines had their own established reference ranges and were available for review. Follow-up data was collected and divided between short-term follow-up (STFU), defined as data collected 2–8 weeks after I-131 treatment, and long-term follow-up (LTFU), defined as data collected 9–24 weeks after I-131 treatment. Cases were excluded from the secondary objective if missing pertinent data, euthanasia prior to post-treatment chemistry, or loss of follow-up.

Normal liver enzyme (NLEZ) activity was defined as all individual LEZ within the established reference ranges established for that analyzer, and ELEZ activity was defined as any individual LEZ above the upper limit of the established reference range for that analyzer.

For the primary objective, chemistry values prior to I-131 treatment for prevalence and observational data were used. For the secondary objective, groups with STFU and LTFU were divided into NLEZ and ELEZ, with NLEZ individuals having no LEZ outside of the reference range and ELEZ individuals having at least one LEZ outside of the upper limits of the reference range for the analyzer used.

### Statistical Analysis

Statistics analysis were calculated using standard statistics software (IBM SPSS build 1.0.0.903). Normality was tested using Shapiro-Wilk test. Normally distributed data are presented in mean (± standard deviation), and non-normally distributed data are presented in median (range).

Comparison of LEZ values at STFU and LTFU was assessed using a Wilcoxon signed rank test. A Kruskal-Wallis test with a 95% confidence interval or Mann-Whitney U test, when appropriate, was used for cats with STFU and LTFU data to determine if there was a significant difference in age, breed, or sex impacting the initial elevated liver enzyme activities prior to I-131 treatment in the reversibility groups. Statistical significance was set as *p* = 0.05.

## Results

### Prevalence and Magnitude of Elevated LEZ

**Figure 1:**
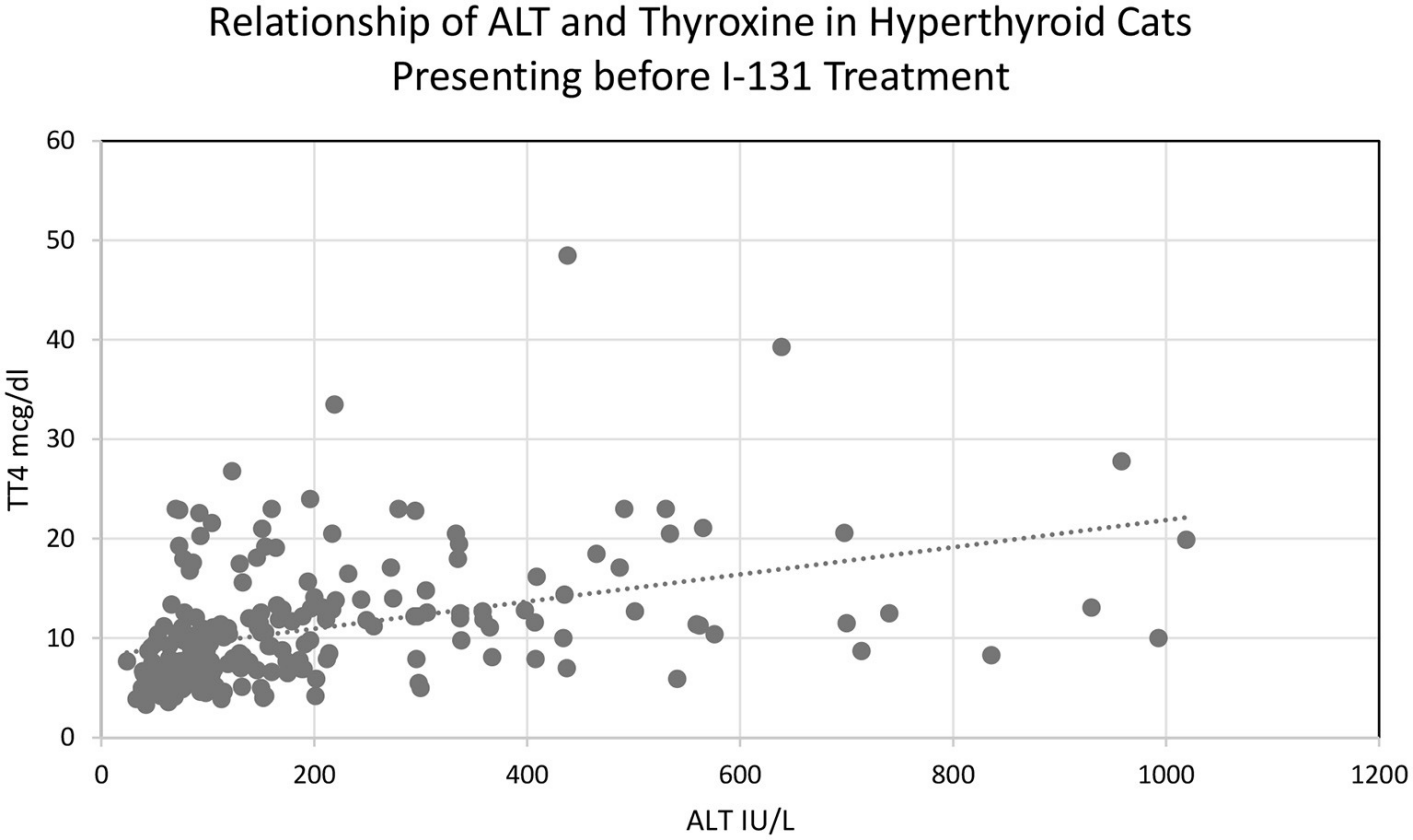
Relationship of ALT and thyroxine concentration in hyperthyroid cats presenting before I-131 treatment.

Two hundred seventeen cats met the inclusion criteria for the primary objective prior to treatment with I-131. The median alanine transaminase (ALT) was 124.0 IU/L (24–1019). ALT was elevated in 123/217 (56.7%) cats. ALT was >500 IU/L in 17 cats (7.8%), whereas 9 (4.1%) had an ALT >700 IU/L and 4 (1.8%) had an ALT >900 IU/L (Table 1). The median alkaline phosphatase (ALP) was 63.00 IU/L (21–454). ALP was elevated in 53/217 (24.4%) cats prior to I-131 treatment. Only 1 cat (0.51%) had an ALP >500 IU/L. The median AST was 41.5 IU/L (16–318 mg/dl; *n* = 186). AST was elevated in 29/217 cats, which also had aspartate aminotransferase (AST) activities elevated (15.59%) (Table 1). All the cats in this study had serum total bilirubin and serum GGT within the reference range based on exclusion criteria. The median serum total bilirubin range was 0.1 mg/dl (0.0–0.7). The median serum GGT was 1.0 IU/L (0.0–10.0). ALT and ALP were positively correlated with thyroxine concentration (Pearson correlation, 2-tailed, *p* < 0.01; Figure 1).

**Table 1:** A summary of liver enzyme activities for cats in cats presenting for I-131 treatment.

**Groups**	**ALT (IU/L)**				**ALP (IU/L)**				**AST (IU/L)**			
	***n***	**Median**	**Mean**	**Range**	***n***	**Median**	**Mean**	**Range**	***n***	**Median**	**Mean**	**Range**
All cats	217	124.0	196.9	24–1,019	215	63.0	81.2	21–454	186	41.5	58.1	16–318
NLEZ Pre	54	72.0	71.6	33–119	53	47.0	50.7	18–96	48	28.0	28.9	16–52
ELEZ Pre	83	214.0	291.4	63–930	80	84.5	106.0	31–586	68	70.0	81.7	22–197
NLEZ STFU	42	35.0	36.9	19–74	42	23.0	25.5	9–59	32	20.0	20.5	10–37
ELEZ STFU	75	53.0	87.5	13–560	75	30.0	42.5	3–341	60	27.0	34.4	13–99
NLEZ LTFU	31	45.0	44.7	19–90	31	21.0	22.9	10–48	24	25.0	25.5	15–38
ELEZ LTFU	48	68.5	76.9	25–231	48	25.5	30.3	8–118	33	35.0	38.4	14–94

*All cats include cats for the primary objective. The secondary objective cats with follow-up data (137) were further divided into normal liver enzyme activities (NLEZ) and elevated liver enzyme activities (ELEZ) with available follow-up 2–8 weeks after treatment (short-term follow-up; STFU) and 9–24 weeks after treatment (long-term follow-up; LTFU). Antech (n = 123) reference ranges: ALT 10–100 IU/L, AST 10–100 IU/L, ALP 6–102 IU/L; IDEXX (n = 73) reference ranges: ALT 27–158 IU/L, AST 16–67 IU/L, ALP 12–59 IU/L*.

### Short-Term and Long-Term Follow-Up

Follow-up after I-131 treatment was available in 137 cats for the secondary objective. Distribution of cases available for follow-up in the ELEZ and NLEZ groups was 83 and 54, respectively. The ELEZ group consisted of 45 spayed females and 38 neutered males. The median age was 12 years (range 8–18 years). Breeds in the ELEZ group included domestic shorthair (71), domestic longhair (5), Balinese (2), Siamese (2), domestic medium hair (1), Maine Coon (1), and American Shorthair (1). The NLEZ group consisted of 27 spayed females and 27 neutered males. The median age was 12 years (range 6–17 years). Breeds in the NLEZ group included domestic shorthair (44), Maine Coon (3), domestic longhair (2), Persian (2), Siamese (1), Angora (1), and Turkish Van (1). Abdominal ultrasound prior to I-131 treatment was performed on 11 cats, 8 in the ELEZ and 3 in the NLEZ groups. Three cats had sonographically normal livers in the ELEZ group. Sonographic hepatic changes included diffuse hepatic hyperechogenicity (*n* = 5), diffuse hepatic hypoechogenicity (*n* = 2), and 1 of each of the following finding: hypoechoic nodules, nodular hepatic cysts, mineralized foci, and suspected fat deposition.

### Impact of I-131 Treatment on Thyroxine Concentration

The median pre-treatment thyroxine concentration in the NLEZ group was 6.8 mg/dl (range 3.06–21.2). The median pre-treatment total thyroxine concentration in the ELEZ group was 11.5 mg/dl (range 4.0–48.5). There was a significant difference in pre-treatment total thyroxine concentration between the two groups (*p* < 0.001). There was no significant difference in total thyroxine concentrations at STFU or LTFU in the NLEZ group (*p* = 0.366) or the ELEZ group (*p* = 0.707).

Overall, the median dose of I-131 administered was 4.0 mCi (2.0–6.5). There was a significant difference in the I-131 dose between groups (*p* = 0.00024), with a median dose of 3.5 mCi (3.2–5.0) for NLEZ compared to 4.0 mCi (2.0–6.5) for ELEZ.

After I-131 treatment, none of the cats in the NLEZ group had persistent hyperthyroidism at follow-up, both STFU and LTFU. In the ELEZ group at STFU, 7 cats had persistent hyperthyroidism, including 6 with ELEZ at that time point, 5/6 (83.33%) with an ALT >100 IU/L, 2/6 (33.33%) with an ALT >500 IU/L, 4/6 (66.67%) with an ALP >100 IU/L, and none with an ALP >500 IU/L. In the ELEZ group at LTFU, 4 cats had persistent hyperthyroidism, while only 2/4 (50%) of the cats had concomitant ELEZ at that time point.

### Short-Term Impact of I-131 Treatment on Liver Enzymes

**Figure 2:**
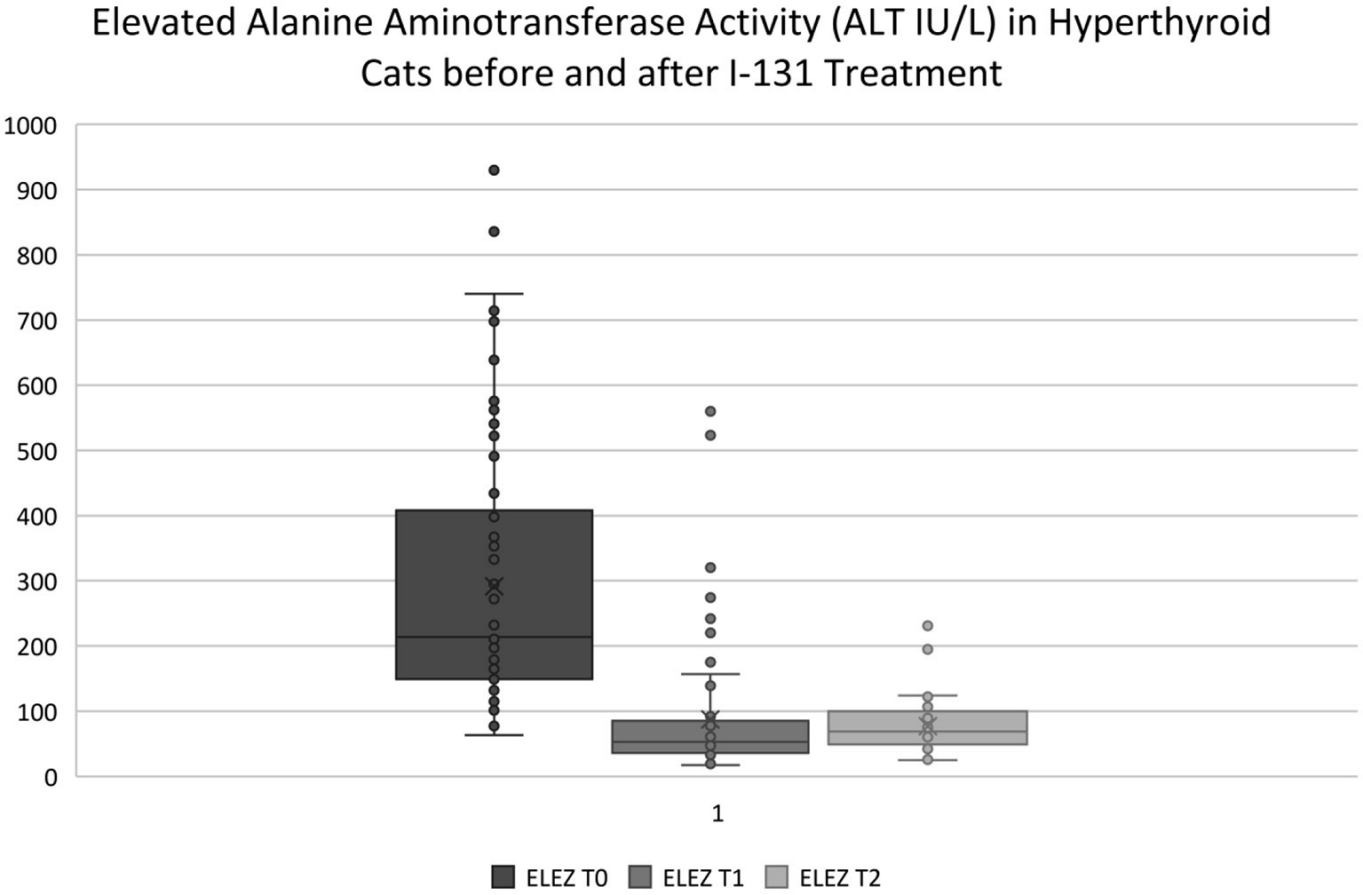
ELEZ T0, Subgroup of hyperthyroid cats presenting for I-131 treatment with elevated ALT activity outside of the reference range; ELEZ T1, Same group of cats with post-op ALT vales at STFU (2–8 weeks); ELEZ T2, Same group of cats with post-op ALT values at LTFU (9–12 weeks). Note: Outliers in the ELEZ T1 and ELEZ T2 data sets are cats that were not successfully treated with I-131 and had persistent elevated ALT activity. All hyperthyroid cats that were successfully treated had ALT activity within the reference range at STFU and LTFU.

Two to 8 weeks after I-131 treatment, the median difference in ALT, ALP, and AST activity for cats in the ELEZ group was 161 IU/L (75.2% decrease; Figure 2), 54.5 IU/L (64.5% decrease), and 43 IU/L (61.4% decrease), respectively. The median difference in ALT, ALP, and AST activity for cats in the NLEZ group was mg/dl 37 IU/L (51.4% decrease), 24 mg/dl (51.1% decrease), and 8.0 (28.6% decrease), respectively. During the STFU timeframe, the decrease in ALT, ALP, and AST was more pronounced in the ELEZ compared to the NLEZ (*p* < 0.001, *p* < 0.001, *p* = 0.007, respectively).

### Long-Term Impact of I-131 Treatment on Liver Enzymes

Nine to 24 weeks after I-131 treatment, the median difference in ALT, ALP, and AST activity for the ELEZ cats at LTFU was 146 IU/L (68.2% decrease; Figure 2), 59.0 mg/dl (69.8% decrease), and 35 IU/L (50% decrease), respectively, compared to baseline before I-131 treatment. The median difference in ALT, ALP, and AST activity for the NLEZ cats at LTFU was 27 IU/L (37.5% decrease), 26 IU/L (55.3% decrease), and 3.0 IU/L (10.7% decrease), respectively. During the LTFU timeframe, the decrease in ALT, ALP, and AST was more pronounced in the ELEZ compared to the NLEZ (*p* < 0.001, *p* = 0.001, and *p* = 0.009, respectively). There was no significant difference between the change in ALT, ALP, and AST from STFU to LTFU between the NLEZ and ELEZ groups (*p* = 0.308, *p* = 0.070, and *p* = 0.116, respectively).

## Discussion

Our study's primary objective revealed that 56.7% proportion of cats presenting for I-131 had elevated liver enzyme activity greater than the reference range for the machine their blood was analyzed on. In total, 123/217 (56.7%) had an elevation in ALT, 53/215 (24.7%) had an elevation in ALP, and 29/186 (15.6%) had an elevation in AST. This finding is lower compared to 75.0–78.9% shown in previous reports with fewer hyperthyroid cats (9, 15). Our study is the first to report the magnitude of ELEZ in a large cohort of cats presenting for I-131 treatment, with ~8% of cats having ALT >500 IU/L. Data from previous publications reporting ELEZ in hyperthyroid cats receiving non-I-131 treatments had markedly fewer cats in each group (8–54 cats), and only mean or median values were provided, and thus, the prevalence of marked elevations in those cohorts was not reported (17–20). Our findings suggest that ELEZ may have a higher amplitude greater than the machine's reference range than previously reported.

Previous studies have shown improvement with the LEZ following non-I-131 therapy, although some of the cats in those studies had persistent ELEZ even with treatment (17–20). It is possible that the observation in these studies may reflect partial improvement of hyperthyroidism or treatment failure rather than non-thyroidal primary hepatic disease, although this data was not reported in the publications. In our study, we observed that cats who failed to achieve a euthyroid state after I-131 had persistent ELEZ, although the magnitude of their median difference in LEZ values significantly improved compared to the pre-treatment values. The decrease in the serum activity of LEZ was observed in both the NLEZ and ELEZ that were successfully treated with I-131.

Our study reports that all cats who were successfully treated with I-131 had liver enzyme activity within the reference range at our STFU and LTFU points. These observations suggest that ELEZ may be a consequence of the hyperthyroid state and likely represent a clinicopathologic phenomenon rather than severe non-thyroidal hepatic disease. In human thyrotoxicosis, liver function test abnormalities have been shown to resolve with euthyroidism and did not correlate with histopathology (14, 21).

Our study showed that the median LEZ value decreased in the ELEZ group at our STFU timepoint. Additionally, the LEZ values had continued to markedly decrease at LTFU in the ELEZ cats, while changes at LTFU were negligible in NLEZ cats. Interestingly, serum activity of LEZ decreased in the NLEZ groups from baseline at the STFU and LTFU timepoints too. This finding suggests that the hyperthyroid state influences liver enzyme activity in most hyperthyroid cats, even when the values are within normal limits. This observation in cats with LTFU suggests that hyperthyroid cats with ELEZ prior to I-131 treatment can require many more weeks to develop NLEZ, perhaps due to more severe hyperthyroidism. This may also reflect decreased osteoblastic activity due to measurement of an isoenzyme in hyperthyroid cats (13). Liver enzyme activity is commonly elevated in untreated thyrotoxicosis in humans but improves after the patients become euthyroid, and the exact mechanism is unclear, while relative hepatic tissue hypoxia secondary to increased oxygen requirement in hepatic and splanchnic circulation is a supporting hypothesis, although pathophysiology studies are lacking to explain the reason (22).

A previous small prospective observational study showed that hyperthyroid cats can have normal liver function and no sonographic abnormalities within their livers despite ELEZ, and all the hyperthyroid cats in that study had NLEZ following I-131 therapy (15). In the present study with a larger cohort of cats, total thyroxine concentration had a positive correlation with liver enzyme activity elevations, and all cats with STFU or LTFU had NLEZ following euthyroidism after successful I-131 treatment. It is reasonable to assume that hyperthyroid cats with ELEZ but normal serum total bilirubin and GGT activity are still good candidates for I-131 therapy, and an extensive diagnostic workup (ex: bile acids testing, abdominal ultrasound, cytology, and/or histopathology) may not be warranted in most cases based on the data presented in the current study.

Individuals in our cohort with higher initial thyroxine concentration had higher ELEZ and were subject to a higher failure rate. Therefore, some cats with marked ELEZ may require higher doses of I-131 than were administered or the need for repeated treatment with I-131. Recent studies have shown that lower doses of I-131 for mild to moderate hyperthyroidism can be used to achieve a euthyroid state and minimize the risk of developing hypothyroidism (7). Our data suggests that the population of NLEZ cats had milder disease when presenting for I-131 treatment compared to ELEZ cats and that ELEZ occur with more advanced hyperthyroidism based on magnitude of serum thyroxine concentration. The magnitude of ELEZ in conjunction with magnitude of thyroxine concentrations and thyroid nodule size on palpation might be a useful marker for more severe hyperthyroidism requiring higher doses of I-131; however, further studies should be done to further assess liver enzyme elevations as an independent variable to help calculate the dose of I-131 in hyperthyroid cats.

There are several limitations to our study. The retrospective nature of the study meant data points were missing and follow-up data was not available for all cats due to owner and veterinarian compliance in performing post-I-131 follow-ups. Similarly, the time range used for measuring post-treatment LEZ was somewhat variable, which is why we chose ranges for the two time points after I-131 was given.

Additionally, the LEZ measurements were done using various diagnostic laboratories and equipment depending on the practice. We therefore elected to use the reference ranges of individual analyzers as an inclusion criterion to overcome this limitation. It is important to note that abnormal values exceeded the machine's respective reference ranges established for that machine analyzer. Indeed, the abnormal values on any analyzer on in-house equipment or when sending to a commercial reference laboratory may preclude a clinician from referral for I-131 or may lead to unnecessary testing for liver function. Therefore, the data presented here is likely useful in clinical practice if each machine's reference ranges are used (i.e., abnormal vs. normal). Ideally, the blood would have been ran on the same blood analyzer machine and then the magnitude of the values and cross-comparison would have been more helpful. Due to the retrospective nature of the study, we had to use the data from the referring veterinarian already submitted; however, there were <6 analyzers used and most of the blood were run on one of two analyzers at two different large commercial laboratory institutions. Therefore, the data is probably accurate and abnormal values can be defined by established reference ranges for that machine and laboratory.

A selection bias may have existed in the cats that were referred for I-131 therapy to our institution, as ill hyperthyroid cats with concurrent primary hepatobiliary disease may have not been considered for referral by the primary veterinarians. Our study did not include the measurement of plasma ammonia concentrations or paired fasted and post-prandial serum bile acid concentrations before and after I-131 treatment, and therefore, hepatic function cannot be assessed in our study; however, a previous study could not demonstrate hepatic dysfunction in hyperthyroid cats presenting for I-131 treatment (15).

In summary, our study demonstrates that ELEZ are common in cats presenting for I-131 treatment, with ALT being the most common ELEZ, and that the prevalence and magnitude of these elevations can be perceived as clinically relevant. Additionally, our study demonstrates that even when ELEZ are markedly elevated prior to I-131 treatment, the liver enzyme activity will likely return to normal after successful resolution of hyperthyroidism using I-131 treatment. This study suggests that I-131 therapy is a safe and effective option in hyperthyroid cats with ELEZ. Although our study did not perform liver function tests, it is possible that true clinically significant primary hepatobiliary disease in hyperthyroid cats presenting for I-131 therapy may be low. Additional diagnostics for liver disease may be necessary if other signs of liver failure are present.

## Data Availability Statement

The raw data supporting the conclusions of this article will be made available by the authors, without undue reservation.

## Ethics Statement

Ethical review and approval was not required for the animal study because this was a retrospective clinical study and therefore exempt from ethical review and approval. Written informed consent for participation was not obtained from the owners because this was a retrospective clinical study and none of the individual patient names or client names are provided in the manuscript and therefore the owners are anonymous.

## Author Contributions

JC is the primary author who collected, compiled, and analyzed the data. He ran the statistics with help from his peers below. He also wrote the manuscript in its entirety with revisions by his mentors and peers. He helped design the initial study with modifications directed by the contributing authors. PC contributed to the revision of the manuscript, helped with the statistics, and read and approved the final manuscript. He was the mentor of JC during his internal medicine residency and during the original study. He is also the residency program leader for Internal Medicine at the hospital. AK helped design the study, helped with multiple revisions of the manuscript during and after JC did his residency, and he read and approved the final manuscript. He is the clinician in charge of the I-131 treatment program for hyperthyroidism at the hospital. He is Chief of Internal Medicine at the hospital. All authors contributed to the article and approved the submitted version.

## Conflict of Interest

The authors declare that the research was conducted in the absence of any commercial or financial relationships that could be construed as a potential conflict of interest.

## Publisher's Note

All claims expressed in this article are solely those of the authors and do not necessarily represent those of their affiliated organizations, or those of the publisher, the editors and the reviewers. Any product that may be evaluated in this article, or claim that may be made by its manufacturer, is not guaranteed or endorsed by the publisher.
